# Immunohistochemical analysis of an ectopic endometriosis in the uterine round ligament

**DOI:** 10.1186/1746-1596-1-27

**Published:** 2006-09-09

**Authors:** Shinichi Terada, Yachiyo Miyata, Hiroaki Nakazawa, Takamitsu Higashimori, Takanari Arai, Yuji Kikuchi, Motohiro Nozaki

**Affiliations:** 1 Division of Plastic Surgery, National Hospital Organization Disaster Medical Center, 3256 Midori-cho, Tachikawa-shi, Tokyo 190-0014, Japan; 2 Department of Plastic and Reconstructive Surgery, Tokyo Women's Medical University, 8-1 Kawada-cho, Shinjuku-ku, Tokyo 162-8666, Japan; 3 Division of Obstetrics and Gynecology, National Hospital Organization Disaster Medical Center, 3256 Midori-cho, Tachikawa-shi, Tokyo 190-0014, Japan

## Abstract

A rare case of the inguinal endometriosis was reported with immunohistochemical analysis. A 28-year-old woman had a thumb-sized tumor in the right groin for two years with a gradual increase in size and pain. An operation revealed an elastic hard tumor with an unclear margin and adhesion to the uterine round ligament. The histology showed irregular proliferation of the endometrial glands and stroma. The glandular epithelium stained weakly positive against CD125 antibody and the stromal matrix stained strongly positive against CD10 antibody. The nucleus in both the epithelial and stromal cells stained strongly positive against progesterone and estrogen receptor antibodies, and the cytoplasm in both types of cells stained moderately positive against COX-2 (cyclooxygenase-2) antibody. In conclusion, the combination of estrogen or progesterone receptor antibody for the nucleus and CD10 or COX-2 antibody for the cytoplasm could enhance the accuracy of diagnosis for ectopic endometriosis.

## Background

Endometriosis is an ectopic occurrence of tissue morphologically and functionally resembling endometrial tissue that is implanted into regions other than the uterus [1]. Although endometriosis occurs most frequently in the intrapelvic organs, many cases of extrapelvic endometriosis throughout the body have been reported. Since Sampson [2] labeled extrauterine adenomyosis as endometriosis, occurrences have been reported not only in intrapelvic tissue including the Douglas fossa, the posterior and anterior cul-de-sacs of the pelvis peritoneum, uterosacral ligaments, the rectum, the colon, and the oviducts, but also in extraperitoneal tissue including the liver [3], the lung [4], and both the cerebral [5] and peripheral nerves [6,7]. Even in extraperitoneal endometriosis, inguinal subcutaneous endometriosis was rarely reported, with an occurrence rate of 0.3~0.8% [1,8-10].

Recent progress in immunohistochemistry has found that CD10 and cyclooxygenase-2 (COX-2) could be important markers for endometrial tissue. Although CD10 is known as a common surface marker of acute lymphoblastic leukemia, it is also expressed in epithelial cells including renal tubular and glomerular cells, breast and salivary gland myoepithelium, prostatic glandular epithelium, and pulmonary alveolar lining cells. However, in endometriosis, CD10 is not expressed in glandular epithelial cells, but in stroma [11,12]. In contrast, COX-2 is a prostaglandin hydroperoxidase, which synthesizes PGH_2 _from PGG_2 _during the processes of inflammation, proliferation, and differentiation, and is expressed in macrophages, fibroblasts, vascular endothelial cells, neurons, and chondrocytes. It is also related to reproductive endometrium, which produces PGE_2 _and PGF_2_α[13,14]. Since we presented an inguinal subcutaneous tumor mass with a postoperative pathological diagnosis of ectopic endometriosis occurring in the uterine round ligament, the purpose of the immunohistochemical analysis in this case report is to compare the stainability of newly applied antibodies to conventional antibodies against CA125, estrogen, and progesterone receptors, to reveal the mechanism of the disease, and to determine the most sensitive procedure for detecting an ectopic endometrial tissue.

## Case report

A 24-year-old female presented a thumb-sized subcutaneous tumor mass in the right side of the pubic region for two years. Because she felt that the tumor size and the pain were gradually increasing, she consulted us for medical care. She had never been pregnant or experienced dysmenorrhea.

Manipulation in the right groin region showed that the mass was located just above the right edge of the pubic tubercle and was a 2 × 3 cm subcutaneous tumor with a slightly rough surface, unclear borderline, and mild tenderness. While no adhesion to the skin and only slight adhesion to the subcutaneous fat tissue were observed, the tumor was firmly attached to the floor without mobility. No remarkable skin region was observed. The laboratory data showed no signs of inflammation with WBC 6400/μl and CRP 0.1 and only slight anemia with Hb 11.8 g/dl. Image analysis of a pelvic CT revealed an irregular subcutaneous mass just above the right edge of the pubic tubercle with the same X-ray absorbance density as that of the muscle. The radiographic diagnosis was that of an inflammatory tumor. Consequently, as the preoperative diagnosis, we considered an inflammatory reaction of a lymph node or a dermoid cyst.

**Figure 1:**
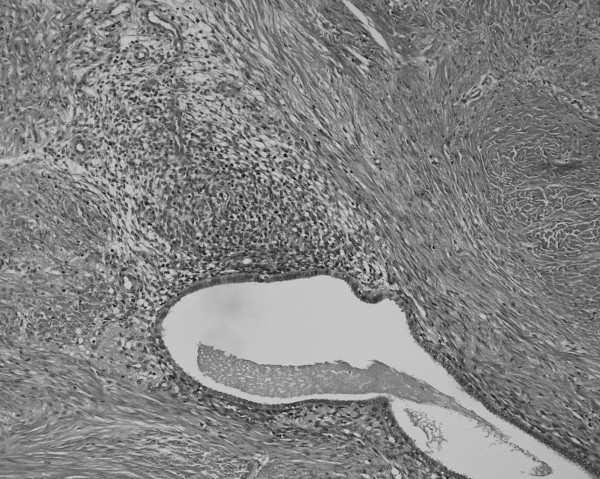
Histopathological view showing irregularly distributed glandular structures and proliferation of stromal cells (H&E stain, ×40).

**Figure 2:**
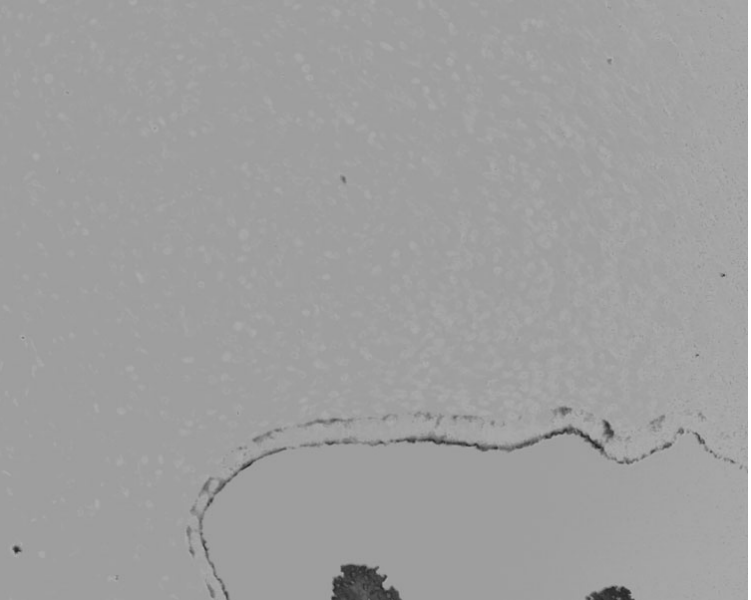
Immunohistochemical analysis of CA125 showing a weak positive stain in the cytoplasm of glandular epithelial cells (×100). The images of immunohistochemical staining were binarized according to brownish (positive area) and light bluish (negative area) colors using Adobe Photoshop.

**Figure 3:**
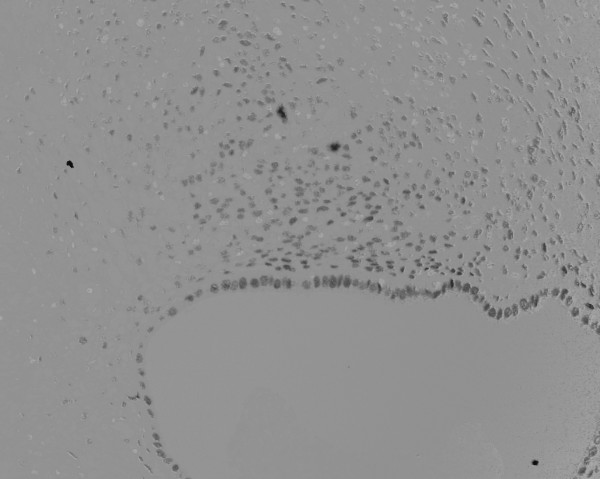
Immunohistochemical analysis of estrogen receptor showing a strong positive stain in the nucleus of the stromal and epithelial cells (×100).

**Figure 4:**
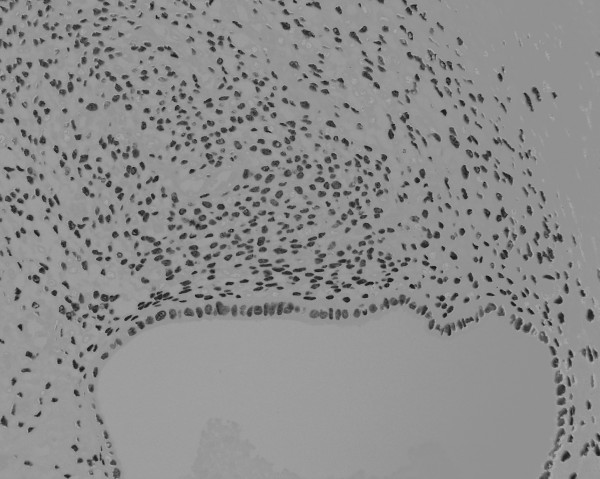
Immunohistochemical analysis of progesterone receptor showing a very strong positive stain in the nucleus of both cells (×100).

**Figure 5:**
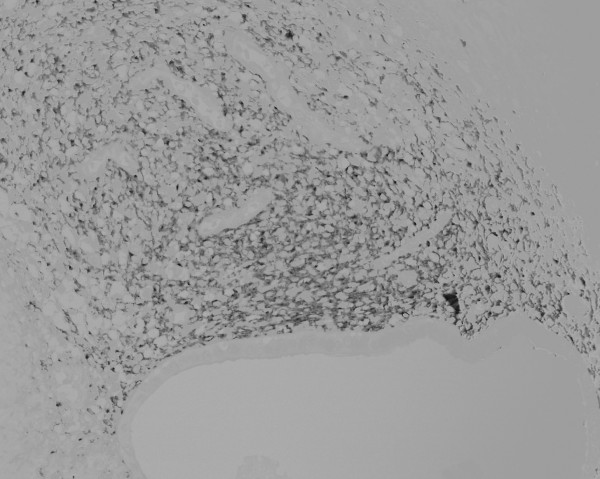
Immunohistochemical analysis of CD10 showing a strong positive stain in the cytoplasm of the stromal cells (×100).

**Figure 6:**
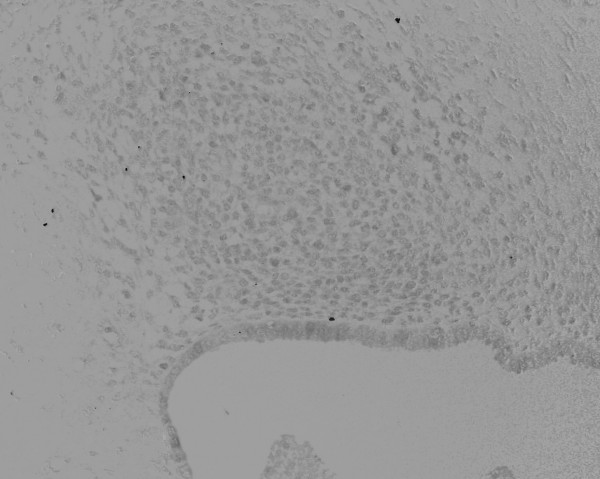
Immunohistochemical analysis of COX-2 showing a moderately positive stain in the cytoplasm of the epithelial cells and the nucleus of both cells (×100).

During the operation, we easily approached the mass through an incision on the medial side of the right groin region. The mass could be manually released from its adhesion to the subcutaneous fat tissue, but was firmly attached to the uterine round ligament with a poorly demarcated borderline. Therefore, we removed the tumor with a part of the uterine round ligament attached. Neither an inguinal hernia nor a sac was observed. From the macroscopic view, fat tissue was attached to the surface of the tumor. The cross section presented a whitish-yellow color with an irregular round shape; the indistinct boundary adhered to the surrounding fat tissue. Small spots containing brownish mucus were observed. The H&E staining of the removed tissue showed several small hollow glands scattered inside the tumor with a stromal structure. The hollow glands were lined with columnar epithelial layers and surrounded by proliferated stromal cells (Fig. 1). Immunohistochemical analyses revealed that antibodies against CA125 (Fig. 2), estrogen receptor (Fig. 3), progesterone receptor (Fig. 4), CD10 (Fig. 5), and COX-2 (Fig. 6) stained positively, but no staining of the CA19-9 antibody was observed. Although five antibodies that we chose had positive staining in the endometrial tissue, there were several differences in the degree of stainability among them. The CA125 antibody stained weakly, mainly in the inner and outer surfaces of the cytoplasm in the glandular endothelial cells, without staining the nuclei of the endothelial and stromal cells. The estrogen receptor, progesterone receptor, and COX-2 antibodies stained both in the stromal and endothelial cells. However, estrogen and progesterone receptor antibodies stained positively only in the nucleus and not the cytoplasm. The estrogen receptor antibody stained more positively in the endothelial cells than in the stromal cells, while the progesterone receptor antibody stained strongly and more positively in the stromal cells than in that of the endothelial cells. COX-2 had stained stronger in the cytoplasm and the nuclei of the endothelial cells than in that of the stromal cells. CD10 stained strongly positive in only the cytoplasm of the stromal cells. These findings indicated that the tumor that had adhered to the uterine round ligament had originated from the endometrial tissue.

After the operation, we consulted an obstetrician and a gynecologist. Neither uterine adenomyosis nor pelvic endometriosis was detected using manipulation, ultrasound examination, and MRI scan. The serum CA125 level was 19.9 U/ml, which is within the normal range. No recurrence of the tumor was observed one year after the operation.

## Discussion

Multiple pathogeneses for the ectopic occurrence of uterine endometriosis have been proposed [15]. They include 1) the endometrium implantation theory based on the reflux of menstrual blood through the oviducts, 2) the coelomic metaplasia theory based on an expression of differential ability in peritoneal mesothelial cells, 3) the lymphatic and vascular metastasis theories of a lymphogenous or hematogenous route, 4) the mechanical transplant theory of remnant endometrium in spontaneous or operative delivery, 5) the embryonic rests theory based on remnants of Wolffian or Mullerian ducts, 6) the composite theory based on a combination of the implantation and metastasis theories, and 7) a recent hypothesis based on the relationship of local immune factors.

The inguinal endometriosis in this case was not related to an inguinal hernia. However, our review of 26 Japanese females including our clinical case and the literature from 1998 through 2003 showed that the initial sites of inguinal endometriosis were the uterine round ligament (50%), the peritoneum of the inguinal hernia sac (23%), and the peritoneum of a femoral hernia (4%). Among these hypotheses, at least two mechanisms including the coelomic metaplasia theory and/or the lymphatic metastasis theory could be involved in inguinal endometriosis.

The clinical features of inguinal endometriosis are summarized from our review as follows. 1) The average age at the first medical visit was 40 years old with the subjects ranging in age from 20 to 56 years old. The average period between the first notice and the first visit to a clinic was 4 years and 1 month, ranging from 1 month to more than 10 years. Since 4 pregnant females and the same number of nonpregnant females were found from a distinct description of pregnancy history, there was no relationship between childbirth and inguinal endometriosis. Sexually mature females, not pregnant, had the most frequent occurrences of inguinal endometriosis.

2) The incidence of inguinal endometriosis according to the right or left side was 22 cases (85%) on the right and 4 cases (15%) on the left. As Jimenez and Miles [9] mentioned, occurrences on the right were significantly more prevalent than were those on the left. Occurrences of thoracic endometriosis syndrome also show the same tendency [16]. Since there is no right and left superiority in inguinal lymph node metastasis of uterine carcinoma, the reason for the right-side superiority in inguinal endometriosis has not yet been discovered.

3) Frequent clinical symptoms were an increase in tumor size, tenderness, and spontaneous pain. As to a relationship with menstrual cycle, 13 cases (50%) were synchronized and the other 13 cases (50%) were not.

4) The most frequent complication was an inguinal hernia (9 cases, 35%). There were unexpectedly rare complications (4 cases, 15%) of uterine adenomyosis and intrapelvic endometriosis. The rest (85%) of the cases occurred independently. Therefore, we consider ectopic endometriosis as an inguinal subcutaneous tumor regardless of cyclic pain and intrapelvic endometriosis.

One of the conventional markers for detecting proliferating endometrial tissue at an ectopic site has been a concentration of CA125 in the serum, but our immunohistochemical analysis revealed that the CA125 antibody stained the weakest in the cytoplasm of glandular epithelial cells. Therefore, other markers should be considered for exploring endometrial tissue. The analyses were summarized as follows: 1) the progesterone receptor antibody showed the strongest positive staining in the nucleus of the stromal cells in comparison to the estrogen receptor, CD10, and COX-2 antibodies; 2) the CD10 antibody had the highest specificity in the cytoplasm of the stromal cells; and 3) the COX-2 antibody had the widest distribution in both the endothelial and stromal cells. Strong positive staining in the nucleus of both cells against the antibodies of the estrogen and progesterone receptors suggested that the tumor had a hormonal responsiveness related to the menstrual cycle the same as did a uterine endometrium. The CD10 antibody had a strong affinity with the cytoplasm of the stromal cells indicating the potential of a diagnostic tool for differentiating from other tumors of epithelial origin. Since COX-2 had the widest distribution in both the endothelial and stromal cells, we speculated that prostaglandins induced by COX-2 were associated with the pain and tenderness of the endometriosis. The combination of the estrogen or progesterone receptor antibody for the nucleus, and the CD10 or COX-2 antibody for the cytoplasm could enhance the accuracy of diagnosis for ectopic endometriosis.

## Summary

A rare ectopic endometriosis in the inguinal subcutaneous region was reported with immunohistochemical analysis. Since our literature review of 26 Japanese females of inguinal endometriosis revealed that 50% of the cases were not related to menstrual cycle, endometriosis of the uterine round ligament should first be considered for differential diagnoses of inguinal subcutaneous tumors in female patients regardless of cyclic pain and intrapelvic endometriosis. The combination of the estrogen or progesterone receptor antibody for the nucleus and the CD10 or COX-2 antibody for the cytoplasm could enhance the accuracy of diagnosis for ectopic endometriosis.

## Authors' contributions

ST, YM, TH and HN carried out the surgery to remove the tumor. TA participated in the design of the immunological analysis. YK and MN participated in the design of the case study and coordination. All authors read and approved the final manuscript.
